# Long-Term Changes in Complete Blood Count-Derived Inflammatory Indices During Risankizumab Treatment for Psoriasis: A 208-Week Multicenter Real-World Study

**DOI:** 10.3390/antib15050097

**Published:** 2026-10-14

**Authors:** Riccardo Balestri, Chiara Pallanch, Michela Magnano, Jacopo Colombini, Giacomo Clarizio, Federico Bardazzi, Vito Di Lernia, Francesca Satolli, Giulia Ganzetti, Cinzia Buligan, Vanessa Mazzoletti, Lucrezia Pacetti, Giulia Odorici, Alessandro Borghi, Rosita Saraceno, Massimiliano Mengarino, Paolo Gisondi, Giampiero Girolomoni, Rossella Lacava, Giulia Rech, Francesco Bellinato

**Affiliations:** 1 Division of Dermatology, Psoriasis Outpatient Service, Azienda Sanitaria Universitaria Integrata del Trentino (ASUIT)—Trento Hospital, P.O. Villa Igea, Via Malta 16, 38121 Trento, Italy; chiara.pallanch@gmail.com (C.P.); rossella.lacava@asuit.tn.it (R.L.); giulia.rech@asuit.tn.it (G.R.); 2 Unit of Dermatology, Lucca Hospital, Azienda USL Toscana Nord Ovest, 55100 Lucca, Italy; michela.magnano@gmail.com (M.M.);; 3 Dermatology Unit, IRCCS Azienda Ospedaliero-Universitaria di Bologna, Policlinico S. Orsola-Malpighi, 40138 Bologna, Italy; giacomoclarizio@gmail.com (G.C.); federico.bardazzi@aosp.bo.it (F.B.); 4 Department of Medical and Surgical Sciences, Alma Mater Studiorum University of Bologna, 40138 Bologna, Italy; 5 Dermatology Unit, Arcispedale Santa Maria Nuova, Azienda USL-IRCCS di Reggio Emilia, 42122 Reggio Emilia, Italy; dilernia.vito@ausl.re.it; 6 Unit of Dermatology, University of Parma, 43126 Parma, Italy; fra.satolli@libero.it; 7 UOC Dermatologia, Ospedale Carlo Urbani, 60035 Jesi, Italy; giulia.ganzetti@alice.it; 8 Institute of Dermatology, Santa Maria della Misericordia University Hospital, 33100 Udine, Italy; 9 Dermatologia, Dipartimento Ospedaliero Oncologico—Medico Specialistico, Azienda Ospedaliero Universitaria di Ferrara, 44124 Ferrara, Italy; pacetti.lucrezia@gmail.com (L.P.); giulai87@hotmail.com (G.O.); alessandro.borghi@unife.it (A.B.); 10 Dermatology and Venereology Unit, Franz Tappeiner Hospital, 39012 Merano, Italy; rositasaraceno@yahoo.it (R.S.);; 11 Department of Medicine, Section of Dermatology, University of Verona, 37126 Verona, Italygiampiero.girolomoni@univr.it (G.G.); francesco.bellinato@gmail.com (F.B.)

**Keywords:** psoriasis, risankizumab, monoclonal antibodies, interleukin-23 (IL-23), neutrophil-to-lymphocyte ratio (NLR), platelet-to-lymphocyte ratio (PLR), monocyte-to-lymphocyte ratio (MLR), systemic immune-inflammation index (SII), systemic inflammation response index (SIRI), body mass index (BMI)

## Abstract

**Background/Objectives**: Blood count-derived inflammatory indices have been proposed as accessible biomarkers of systemic inflammation in psoriasis, but their longitudinal behavior during selective interleukin-23 blockade remains uncertain. We evaluated these indices in psoriasis patients treated with risankizumab. **Methods:** We conducted a multicenter retrospective real-world study (DERMIS), including adults with cutaneous psoriasis, with or without psoriatic arthritis, treated with risankizumab across 10 Italian centers, from national reimbursement up to 1 December 2025. Psoriasis Area and Severity Index (PASI) and complete blood count-derived indices—neutrophil-to-lymphocyte ratio (NLR), platelet-to-lymphocyte ratio (PLR), monocyte-to-lymphocyte ratio (MLR), systemic immune-inflammation index (SII), and systemic inflammation response index (SIRI)—were assessed at baseline and at weeks 12, 24, 52, 104, 156, and 208. Analyses used available data and sensitivity cohorts with follow-up at weeks 52, 104, and 208. **Results**: Among 250 patients, 43 had data at week 208. PASI scores decreased significantly at all follow-up time points. In contrast, systemic indices showed heterogeneous, non-linear trajectories: NLR, SII, and SIRI fluctuated without consistent sustained reductions, whereas MLR remained broadly stable. PLR showed the most reproducible downward trend, with nominal reductions at weeks 24 and 52; after Bonferroni correction across the six repeated visits within PLR, the reduction at week 52 remained statistically significant, whereas the week-24 reduction did not. Body mass index (BMI)-related differences were inconsistent and did not remain significant after Bonferroni correction. Sex, cardiometabolic comorbidities, and previous biological exposure showed no consistent effect. **Conclusions**: These findings challenge the use of routine blood count-derived indices as reliable biomarkers of cutaneous response to risankizumab.

## 1. Introduction

Psoriasis is currently recognized as a chronic immune-mediated systemic disease rather than a purely cutaneous disorder. The clinical phenotype is dominated by erythematous and scaly plaques, but the disease process involves sustained immune activation and is frequently associated with psoriatic arthritis, cardiovascular disease, metabolic syndrome, diabetes, obesity, non-alcoholic fatty-liver disease, and psychological burden [1,2,3,4,5]. The interleukin (IL)-23/T helper 17 (Th17) axis is central to psoriatic inflammation. Selective inhibition of IL-23, including blockade of the p19 subunit by risankizumab, has reshaped the therapeutic landscape of moderate-to-severe plaque psoriasis by offering high rates of durable skin clearance [6,7,8].

The increasing availability of monoclonal antibodies for psoriasis has also changed the expectations placed on treatment monitoring. Conventional clinical scores, particularly the Psoriasis Area and Severity Index (PASI), remain the most widely used measures of cutaneous response. However, PASI is operator-dependent and primarily reflects visible skin disease. It may not fully capture the systemic inflammatory burden of psoriatic disease or the inflammatory background generated by cardiometabolic comorbidities [3,4,9]. For this reason, there is growing interest in inexpensive, reproducible, and routinely available biomarkers that might complement clinical assessment during biologic therapy.

Blood count-derived inflammatory indices have emerged as candidate markers of systemic inflammation. The neutrophil-to-lymphocyte ratio (NLR), platelet-to-lymphocyte ratio (PLR), monocyte-to-lymphocyte ratio (MLR), systemic immune-inflammation index (SII), and systemic inflammation response index (SIRI) combine different components of innate immunity, adaptive immunity, and platelet-mediated inflammation. Several studies have reported higher NLR, PLR, MLR, SII, or SIRI in patients with psoriasis than in controls, and some have shown correlations with PASI, psoriatic arthritis, or disease severity [10,11,12,13,14,15]. Population-based analyses have also linked SII and related indices with psoriasis risk, although these associations are often non-linear and sensitive to metabolic covariates [16,17].

Despite this biological plausibility, the clinical usefulness of these indices during biological treatment remains uncertain. Retrospective and prospective series have reported decreases in NLR, PLR, MLR, SII, and SIRI after systemic or biological therapy, but published cohorts are heterogeneous with regard to treatment class, duration of follow-up, sample size, disease phenotype, and comorbidity burden [18,19,20,21,22,23]. Data specific to IL-23 inhibition are particularly limited. A 52-week prospective multicenter study of risankizumab in responder patients suggested a reduction in NLR, SII, and SIRI, especially among very early responders [18]. In 120 patients with moderate-to-severe psoriasis treated over 12 months, risankizumab achieved substantial clinical response (55% PASI 0 clearance); however, systemic inflammatory markers (SII and SIRI) exhibited inconsistent fluctuations that did not parallel the observed clinical improvement [24]. A post hoc analysis of randomized guselkumab trials showed reductions in NLR, PLR, and MLR versus placebo, while a longer-term real-world study of guselkumab reported weak correlations between inflammatory indices and skin severity, supporting a partial dissociation between cutaneous and systemic inflammation [25,26].

The Dynamic Effects of Risankizumab on Markers of Inflammation and Skin (DERMIS) study was designed to address these gaps. The study focuses on a single monoclonal antibody, risankizumab, in a large multicenter Italian real-world cohort, with a follow-up extending to 208 weeks. The primary objective was to evaluate the longitudinal trend and clinical relevance of NLR, PLR, MLR, SII, and SIRI in patients with psoriasis treated with risankizumab. Secondary objectives were to explore whether body mass index (BMI), sex, cardiometabolic comorbidities, previous biological exposure, and cutaneous response influenced these indices over time.

## 2. Materials and Methods

### 2.1. Study Design and Setting

DERMIS is a multicenter retrospective observational study conducted across 10 Italian public, hospital-based dermatology centers. Patients were enrolled between 4 March 2020 (the date of national reimbursement for risankizumab in Italy) [27] and 1 December 2025.

This study aims to evaluate the long-term real-world effects of risankizumab on blood count-derived inflammatory indices in patients with psoriasis and to identify clinical factors associated with longitudinal trend in these parameters.

Institutional Review Board approval was waived since the study procedures did not deviate from standard clinical practice. The study was conducted in accordance with the 1964 Declaration of Helsinki and its subsequent amendments. Data collection and management complied with all applicable regulations regarding patient protection and privacy. Reporting adheres to the STROBE (Strengthening the Reporting of Observational Studies in Epidemiology) guidelines.

### 2.2. Participants

Eligible patients were adults aged 18 years or older with plaque psoriasis, with or without psoriatic arthritis, who had initiated risankizumab in routine clinical practice and had available baseline clinical and laboratory data. No minimum duration of risankizumab exposure was required for inclusion; patients contributed data only for the follow-up visits they attended and for which measurements were available. Previous biologic therapy was permitted and recorded for subgroup analyses. Given the retrospective, real-world nature of the study, no study-specific washout period from previous biologic therapies was required beyond that routinely applied by treating clinicians in clinical practice. Exclusion criteria included complete blood count abnormalities attributable to active infectious, neoplastic, or hematological conditions, as well as inflammatory diseases other than psoriasis or psoriatic arthritis. Patients with incomplete follow-up data were retained in the study, and analyses were conducted using all available observations for each variable at each visit (available-case analysis).

### 2.3. Variables and Follow-Up

At baseline, the electronic Case Report Form captured age, sex, weight, height, BMI, disease duration, comorbidities, PASI, white blood cell count, absolute neutrophil count, lymphocyte count, monocyte count, platelet count, biologic-naive or biologic-experienced status, and the date of risankizumab initiation. PASI and complete blood count data were collected at baseline and at 12, 24, 52, 104, 156, and 208 weeks. Treatment failure, discontinuation, and loss to follow-up were also recorded.

The inflammatory indices were calculated as follows: NLR = neutrophils/lymphocytes; PLR = platelets/lymphocytes; MLR = monocytes/lymphocytes; SII = (neutrophils × platelets)/lymphocytes; and SIRI = (neutrophils × monocytes)/lymphocytes. BMI was categorized as normal weight or underweight (<25.0 kg/m^2^), overweight (25.0 to <30.0 kg/m^2^), and obese (≥30.0 kg/m^2^). Additional subgroup analyses were conducted based on sex, diabetes status, cardiovascular comorbidities (coronary artery disease and/or hypertension), previous biological exposure, psoriatic arthritis and special-site involvement (e.g., nail psoriasis and genital psoriasis).

### 2.4. Endpoints

The primary endpoint was the longitudinal trend in NLR, PLR, MLR, SII, and SIRI over the course of risankizumab treatment. Secondary endpoints included: the influence of BMI, sex, diabetes, coronary artery disease and hypertension, as well as previous biological exposure and special-site involvement on inflammatory-index trajectories; long-term clinical efficacy as measured by PASI; correlations between PASI and inflammatory indices. A responder sub-analysis compared patients achieving PASI 90 by week 24 with those achieving PASI 90 at a later time point.

### 2.5. Statistical Analysis

Continuous variables were summarized using the number of observations, missing values, mean with standard deviation (SD), and median. For each post-baseline visit, changes from baseline were calculated exclusively for subjects with available data at both baseline and the corresponding visit. Within-subject changes from baseline were assessed using paired *t*-tests. Between-group comparisons at each time point were performed using independent-sample *t*-tests, assuming approximate normality of distributions, whereas comparisons across the three BMI categories were performed using the Kruskal–Wallis test. Correlations were evaluated using Spearman’s rank correlation coefficients. All tests were two-sided, and *p*-values were interpreted descriptively as measures of temporal change or between-group differences. To account for multiple testing, the Bonferroni correction was applied to the six comparisons performed across follow-up visits, separately for each parameter and analysis. Statistical significance after correction was defined as an adjusted *p*-value < 0.05. To limit potential bias arising from the progressive reduction in sample size during long-term follow-up, sensitivity analyses were conducted for patients with data available to weeks 52, 104, and 208. Analyses were performed using SAS^®^ software (version 9.4; SAS Institute Inc., Cary, NC, USA).

## 3. Results

### 3.1. Study Population

**Figure 1:**
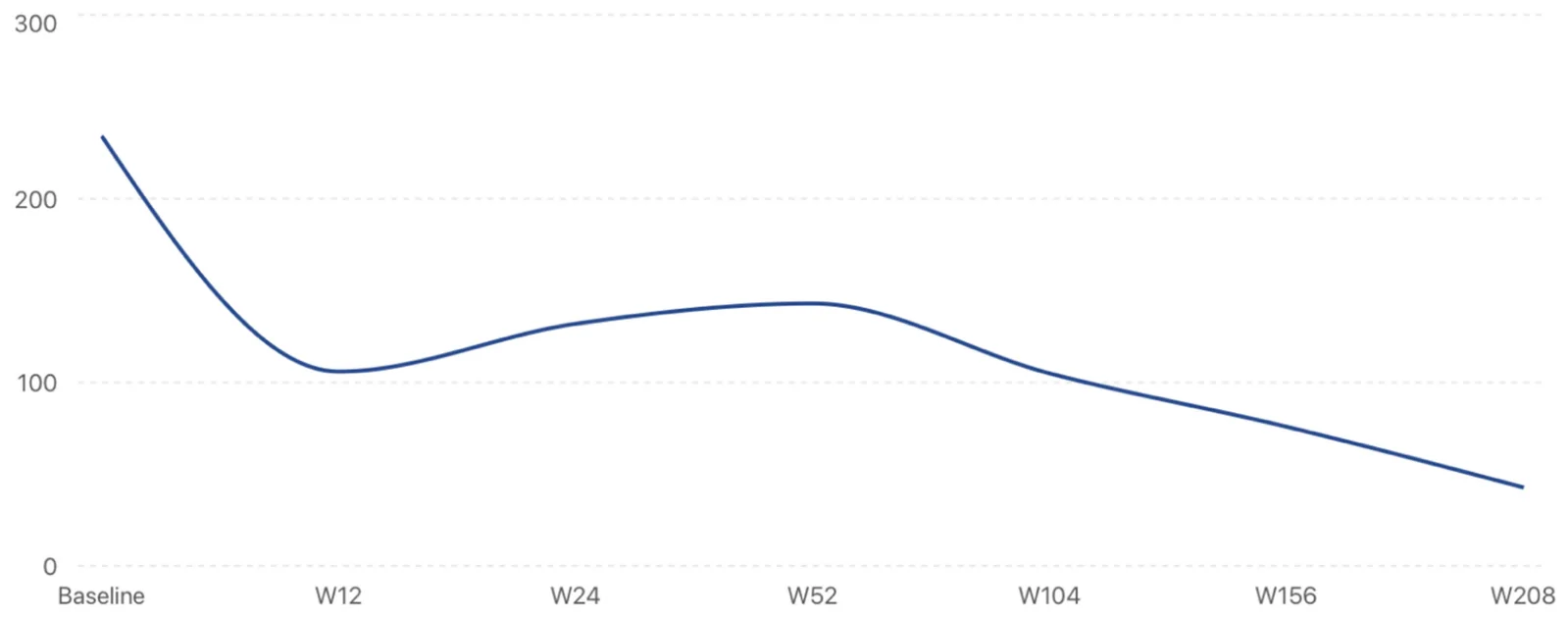
Trend over time in the number of subjects with available values for NLR, MLR, PLR, SII, and SIRI during follow-up. The Y-axis reports the total number of patients, while the X-axis shows time in weeks.

The study included 250 patients. Data availability progressively declined over time, mainly because many patients had initiated risankizumab more recently and had not yet reached the later follow-up visits. Available inflammatory-index observations included 234 patients at baseline, 107 at week 12, 132 at week 24, 143 at week 52, 105 at week 104, 76 at week 156, and 43 at week 208 (Figure 1). Treatment was discontinued in 21 patients: 9 due to therapeutic ineffectiveness (6 with primary non-response and 3 with secondary loss of efficacy), 5 due to adverse events or subsequent contraindications (onset of joint pain, cancer diagnosis, or pregnancy), 6 due to loss to follow-up, and 1 due to a treatment-unrelated death.

Baseline demographic and clinical characteristics are summarized in Table 1. Women accounted for 60.2% of the cohort (n = 150) and men for 39.8% (n = 99), and sex was unavailable for one patient. Age ranged from 22 to 89 years, with a mean of 56.6 ± 14.5 years and a median of 56.5 years.

### 3.2. Overall Longitudinal Trend in Inflammatory Indices

**Figure 2:**
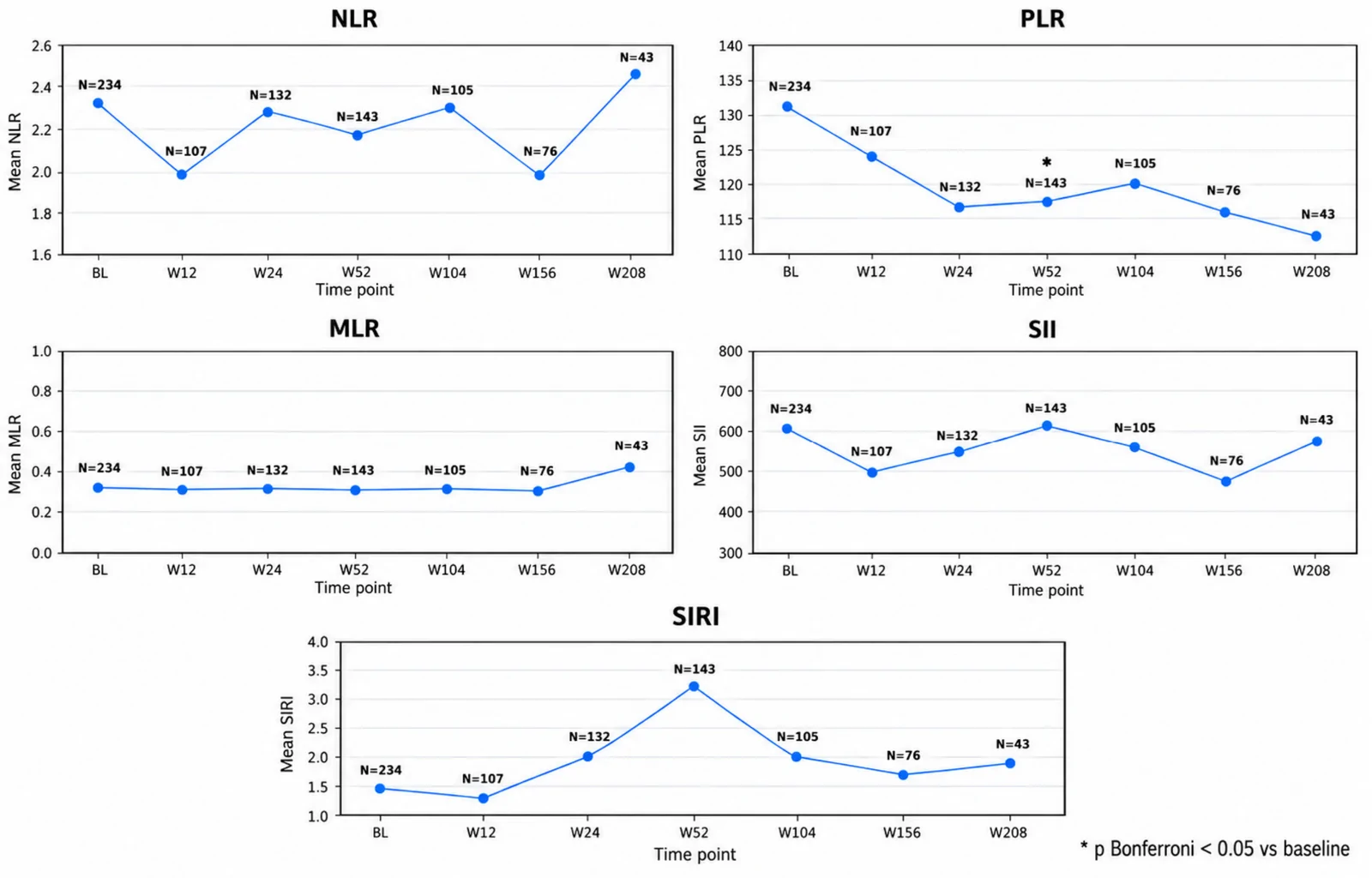
Longitudinal trajectories of systemic inflammatory indices during risankizumab treatment.

Overall, the five blood count-derived inflammatory indices showed heterogeneous longitudinal changes during follow-up (Table 2, Figure 2). NLR and SII displayed fluctuating trends without significant changes from baseline. MLR remained remarkably stable throughout the observation period, with no significant longitudinal variation. In contrast, PLR showed an overall downward trajectory, with nominal reductions at weeks 24 (*p* = 0.0209) and 52 (*p* = 0.0040). After Bonferroni correction across the six repeated visits within PLR, the week-52 reduction remained statistically significant (adjusted *p* = 0.0239), whereas the week-24 reduction did not (adjusted *p* = 0.1252). SIRI showed a nominal increase at week 24 (*p* = 0.0221), which did not remain significant after correction (adjusted *p* = 0.1329). No other longitudinal comparison was significant after Bonferroni adjustment.

### 3.3. Sensitivity Analyses by Follow-Up Availability

Among patients with available data through week 52 (n = 142), longitudinal changes were heterogeneous. NLR showed a modest early reduction followed by a return towards baseline, whereas PLR declined through week 24 and remained below baseline at week 52. The PLR reductions at weeks 24 and 52 remained statistically significant after Bonferroni correction (adjusted *p* = 0.0391 and 0.0239, respectively). MLR remained stable, while SII and SIRI decreased at week 12, rose at week 24, and returned to values close to baseline by week 52. The nominal increase in SIRI at week 24 did not remain significant after correction (adjusted *p* = 0.1576).

Findings were broadly similar among patients with available data through week 104 (n = 104). NLR showed only modest fluctuations, PLR remained generally below baseline, despite a non-linear trajectory, and MLR was stable. SII and SIRI fluctuated over time, with SII exceeding its baseline value at week 104. The PLR reduction at week 52 remained statistically significant after Bonferroni correction (nominal *p* = 0.0049; adjusted *p* = 0.0294), whereas the nominal SIRI increase at week 24 did not (adjusted *p* = 0.2447).

Among patients with available data through week 208 (n = 43), NLR, SII, and SIRI exhibited fluctuating patterns, with values at week 208 slightly above baseline for NLR and SII and more markedly above baseline for SIRI. PLR remained lower than baseline at four years despite a non-linear course, whereas MLR remained stable. No statistically significant longitudinal changes were observed in this subgroup.

### 3.4. BMI, Sex, Cardiometabolic Comorbidities, and Biological Exposure

#### 3.4.1. BMI and Inflammatory Indices (Table 3, Figure 3)

Inflammatory-index trajectories varied across BMI categories, with frequent overlap between groups and no consistent between-group differences during follow-up. Nominal differences were observed at selected time points, including week 208 for MLR (*p* = 0.0247) and SIRI (*p* = 0.0289), but neither remained statistically significant after Bonferroni correction across the six repeated visits within the corresponding parameter (adjusted *p* = 0.1480 and 0.1733, respectively). No BMI-related comparison remained significant after adjustment.

**Table 3:** Longitudinal changes in systemic inflammatory indices stratified by BMI category during risankizumab treatment.

	BMI Category	Baseline	Week 12	Week 24	Week 52	Week 104	Week 156	Week 208
**NLR** **Neutrophil-to-lymphocyte ratio**	Normal weight	2.0 (1.3–2.7)	1.8 (1.2–2.7)	1.8 (1.6–2.4)	2.0 (1.4–2.8)	1.8 (1.4–3.0)	1.8 (1.4–2.6)	2.2 (1.7–2.8)
	Overweight	1.8 (1.3–2.8)	1.6 (1.2–1.9)	1.7 (1.4–2.5)	1.5 (1.3–2.2)	1.5 (1.3–1.9)	1.6 (1.3–2.1)	2.6 (1.6–2.8)
	Obesity	2.3 (1.7–2.7)	2.0 (1.5–2.4)	2.1 (1.5–2.9)	2.0 (1.5–2.4)	2.1 (1.7–2.6)	1.7 (1.6–2.4)	1.5 (1.3–2.3)
	Across BMI *p*-value nominal *	-	0.512	0.568	0.148	0.378	0.128	0.080
	Across BMI *p*-value Bonferroni **	-	1.000	1.000	0.885	1.000	0.770	0.481
**PLR** **Platelet-to-lymphocyte ratio**	Normal weight	114.9 (92.4–150.6)	109.9 (87.8–141.2)	110.4 (78.7–141.5)	112.3 (88.0–156.4)	114.8 (86.2–149.7)	118.6 (96.8–148.7)	145.6 (106.0–158.1)
	Overweight	116.1 (95.3–149.0)	104.7 (79.2–144.3)	122.3 (91.0–150.6)	104.1 (77.6–136.9)	104.8 (81.3–127.8)	102.8 (76.9–126.5)	106.8 (91.0–125.1)
	Obesity	117.5 (93.9–159.2)	112.7 (93.0–136.5)	102.8 (79.4–169.3)	101.7 (83.9–131.0)	116.2 (90.5–150.5)	121.3 (94.1–147.0)	78.4 (59.5–97.3)
	Across BMI *p*-value *	-	0.977	0.716	0.663	0.928	0.774	0.318
	Across BMI *p*-value Bonferroni **	-	1.000	1.000	1.000	1.000	1.000	1.000
**MLR** **Monocyte-to-lymphocyte ratio**	Normal weight	0.3 (0.2–0.3)	0.3 (0.2–0.4)	0.2 (0.2–0.3)	0.3 (0.2–0.3)	0.3 (0.2–0.3)	0.2 (0.2–0.3)	0.3 (0.2–0.4)
	Overweight	0.3 (0.2–0.3)	0.2 (0.2–0.3)	0.3 (0.2–0.4)	0.2 (0.2–0.3)	0.2 (0.2–0.3)	0.2 (0.2–0.3)	0.4 (0.3–0.6)
	Obesity	0.3 (0.3–0.4)	0.3 (0.2–0.3)	0.3 (0.2–0.4)	0.3 (0.2–0.3)	0.3 (0.2–0.4)	0.3 (0.2–0.4)	0.3 (0.2–0.3)
	Across BMI *p*-value * vs. baseline	-	0.145	0.610	0.171	0.905	0.599	**0.025**
	Across BMI *p*-value Bonferroni **	-	0.871	1.000	1.000	1.000	1.000	0.148
**SII** **Systemic immune-inflammation index**	Normal weight	484.4 (327.9–666.7)	441.2 (299.1–740.5)	450.1 (314.5–680.4)	485.6 (327.3–762.0)	504.0 (353.4–717.8)	441.9 (318.6–647.0)	538.4 (368.6–861.5)
	Overweight	448.1 (342.6–733.9)	391.3 (278.1–523.3)	460.6 (341.2–675.0)	415.0 (321.4–537.1)	366.7 (293.9–494.9)	417.0 (328.8–481.3)	564.5 (356.6–633.1)
	Obesity	528.9 (417.6–633.8)	438.6 (328.4–610.6)	481.9 (328.4–697.9)	439.8 (323.1–635.0)	489.0 (414.0–607.4)	420.0 (339.6–678.3)	335.3 (234.9–499.5)
	Across BMI *p*-value * vs. baseline	-	0.855	0.361	0.199	0.309	0.133	0.131
	Across BMI *p*-value Bonferroni **	-	1.000	1.000	1.000	1.000	0.797	0.788
**SIRI** **Systemic inflammation response index**	Normal weight	1.0 (0.7–1.4)	1.3 (0.7–1.6)	1.2 (0.8–1.6)	1.2 (0.8–1.9)	1.1 (0.9–1.7)	1.0 (0.6–1.7)	1.2 (0.8–1.7)
	Overweight	1.0 (0.7–1.7)	0.8 (0.7–1.1)	1.0 (0.6–2.1)	0.9 (0.6–1.5)	0.8 (0.6–1.3)	0.9 (0.7–1.4)	2.2 (1.5–2.4)
	Obesity	1.4 (1.1–1.8)	1.2 (0.7–1.5)	1.3 (0.9–1.9)	1.2 (0.8–1.9)	1.3 (0.7–1.9)	1.0 (0.8–1.9)	1.2 (0.8–2.1)
	Across BMI *p*-value * vs. baseline	-	0.122	0.541	0.212	0.353	0.052	**0.029**
	Across BMI *p*-value Bonferroni **	-	0.732	1.000	1.000	1.000	0.314	0.173

Data are presented as median (Q1–Q3). BMI categories were defined as normal weight (BMI < 25 kg/m^2^), overweight (BMI 25–<30 kg/m^2^), and obesity (BMI ≥ 30 kg/m^2^). * Kruskal–Wallis *p*-values indicate whether changes from baseline differed significantly across the three BMI categories. ** For each parameter, the Bonferroni correction was applied to the six comparisons against baseline. Statistically significant *p*-values are shown in bold. Abbreviations: BMI, body mass index.

Among patients with available data through week 52 (n = 142), NLR, SII, and SIRI increased from baseline in normal-weight patients but remained essentially stable or showed a slight variation in overweight patients and patients with obesity. MLR remained stable across all BMI groups, whereas PLR tended to decrease irrespective of BMI. No significant between-group differences were observed.

Among patients with available data through week 104 (n = 104), inflammatory indices remained variable, with generally higher values in patients with obesity at most time points. MLR remained stable, whereas NLR, PLR, SII, and SIRI showed modest fluctuations. None of the between-group comparisons reached statistical significance.

Among patients with available data through week 208 (n = 43), all indices showed variable trajectories with frequent overlap between BMI groups. No statistically significant difference was observed after Bonferroni correction.

**Figure 3:**
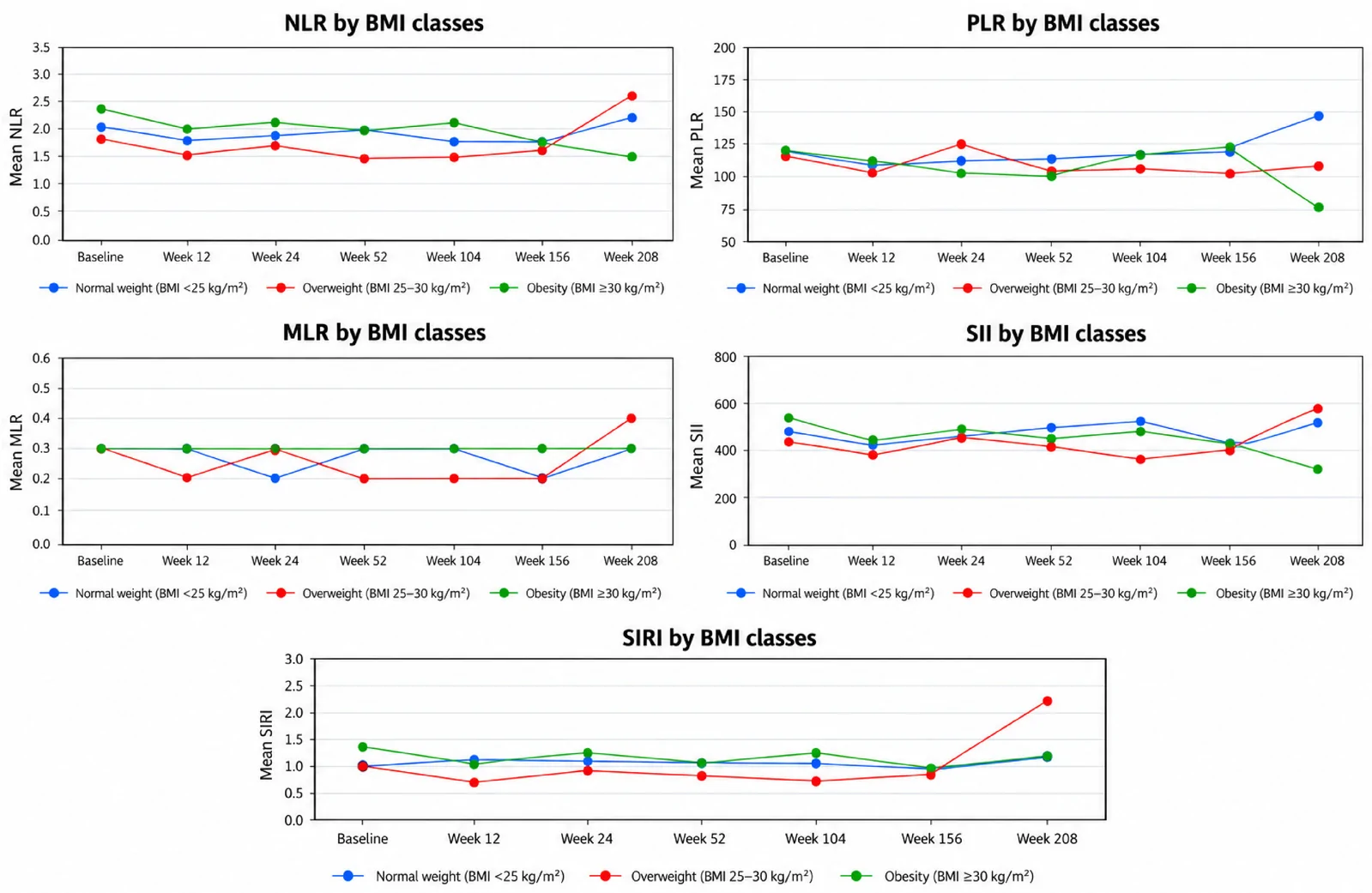
Longitudinal trajectoriesin systemic inflammatory indices stratified by BMI category during risankizumab treatment.

#### 3.4.2. Sex and Inflammatory Indices

Longitudinal changes in inflammatory indices were broadly similar in men and women, with no consistent sex-related differences during follow-up. At week 208, nominal between-sex differences were observed for NLR (*p* = 0.0216), PLR (*p* = 0.0036), and SII (*p* = 0.0110). After Bonferroni correction across the six repeated visits within each parameter, only the PLR difference remained statistically significant (adjusted *p* = 0.0214); the NLR and SII findings did not (adjusted *p* = 0.1298 and 0.0661, respectively). At this time point, PLR showed a more marked reduction from baseline in men. These late differences were not supported by a consistent pattern at earlier assessment points.

#### 3.4.3. Diabetes and Inflammatory Indices

At baseline, patients with diabetes tended to have higher NLR, PLR, SII and SIRI values than those without diabetes. However, longitudinal trends were broadly comparable between groups, and MLR remained similar throughout follow-up. No statistically significant differences between groups were observed.

#### 3.4.4. Cardiovascular Comorbidities and Inflammatory Indices

Patients with coronary artery disease and hypertension tended to have higher mean inflammatory-index values at several time points. Nevertheless, longitudinal changes in NLR, PLR, MLR, SII, and SIRI were comparable between patients with and without cardiovascular comorbidities, with no significant differences between groups.

#### 3.4.5. Previous Exposure to Biological Therapy

No significant differences in the longitudinal trend of NLR, PLR, MLR, SII, or SIRI was observed between biologic-naive and biologic-experienced patients. Changes from baseline were generally modest and highly variable in both groups.

### 3.5. PASI Response (Figure 4)

Baseline PASI score was available for 234 patients (mean value ± SD: 12.8 ± 7.3, range: 0—53). PASI score decreased markedly during the first 12 weeks, showed a further smaller reduction by week 24, and remained broadly stable thereafter through week 208. Reductions from baseline were statistically significant at every follow-up visit (all *p* < 0.01).

**Figure 4:**
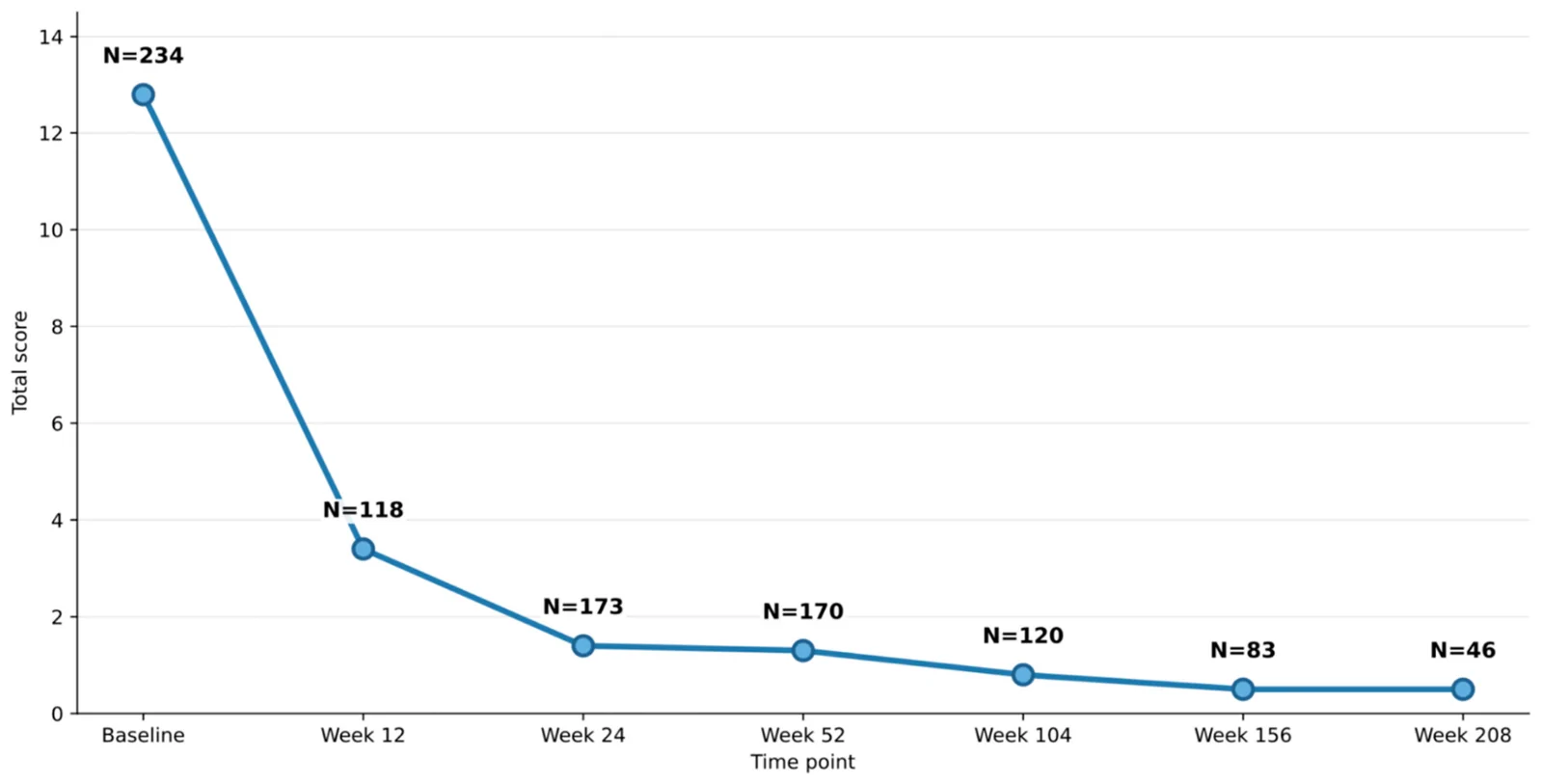
Trend over time in mean PASI values among patients with available data. PASI values are plotted on the Y-axis and time in weeks on the X-axis. Numbers adjacent to each data point indicate the sample size (N) available for parameter calculation at that specific time point.

#### 3.5.1. Relationship Between PASI and Inflammatory Indices

Baseline inflammatory indices were not significantly correlated with PASI, with correlation coefficients close to zero for all markers. Likewise, no significant differences in inflammatory-index trajectories were observed between early responders, who achieved PASI 90 by week 24 and late responders, who achieved it later.

#### 3.5.2. BMI and PASI (Figure 5)

Patients with obesity consistently had higher mean PASI values than normal-weight and overweight patients at baseline and throughout follow-up. Between-group differences reached statistical significance only at weeks 24 and 156, when PASI values were lower in normal-weight and overweight patients than in those with obesity. At the remaining time points, PASI changes did not differ significantly across BMI categories.

**Figure 5:**
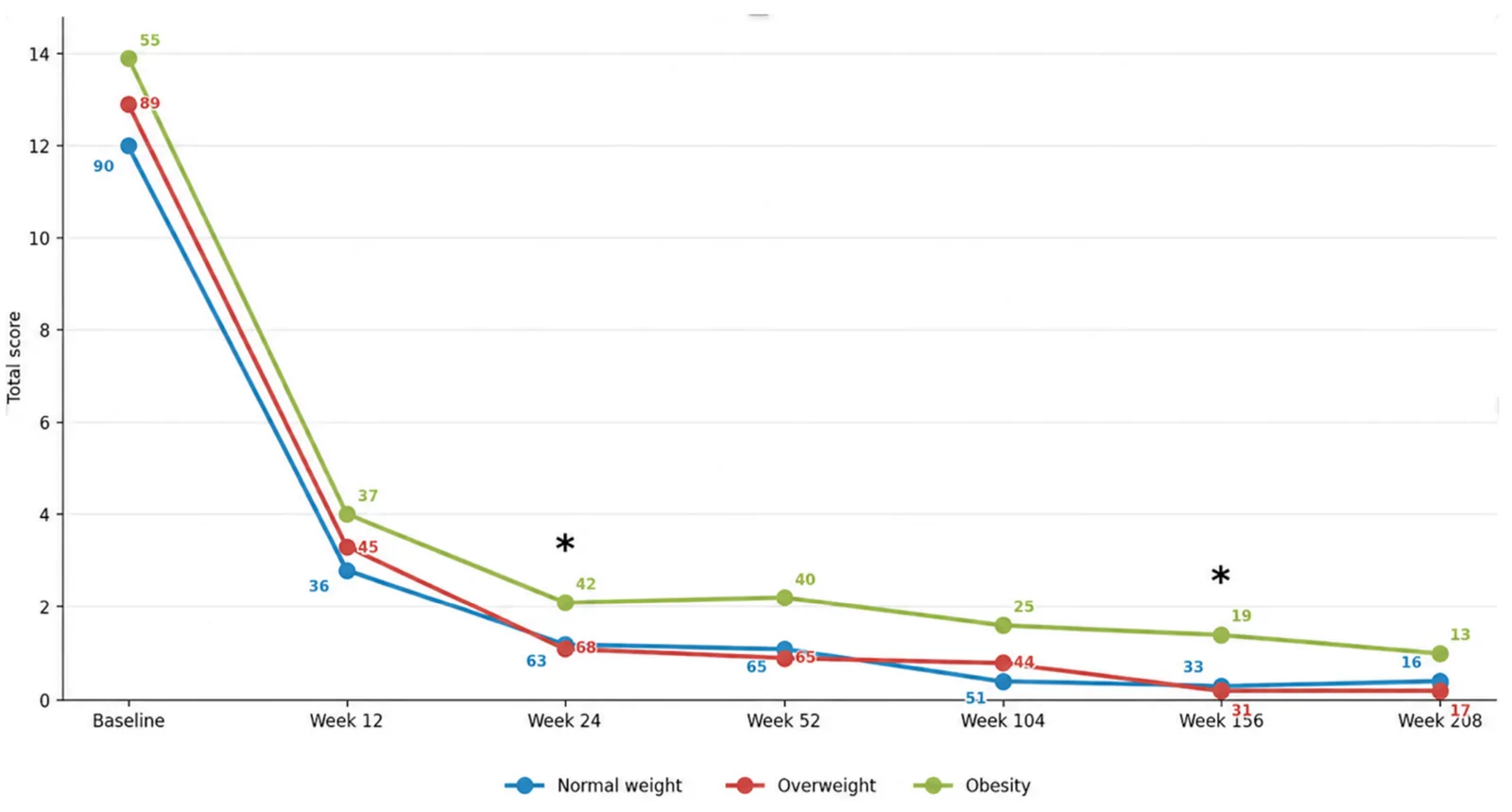
Trend over time in mean PASI values stratified by BMI category. PASI values are plotted on the Y-axis and time in weeks on the X-axis. Numbers adjacent to each data point indicate the sample size (N) available for PASI calculation at that specific time point within the corresponding BMI class. Asterisks (*) denote statistically significant differences (*p* < 0.05).

### 3.6. Longitudinal Changes According to Psoriatic Arthritis and Special-Site Involvement (Table 4 and Table 5)

#### 3.6.1. Psoriatic Arthritis

Among patients with psoriatic arthritis, the longitudinal trends of NLR, MLR, SII, and SIRI were broadly comparable to those observed in patients without arthritis, with no significant between-group differences in changes from baseline. PLR progressively decreased through week 156 and subsequently returned towards baseline by week 208. At week 208, a nominal difference in PLR change in PLR was observed between patients with and without psoriatic arthritis, with a mean increase of 5.1 in the former and a mean decrease of 36.0 in the latter (*p* = 0.036). However, the absence of a similar signal at earlier time points does not support a specific or persistent trajectory associated with psoriatic arthritis.

**Table 4:** Longitudinal changes in systemic inflammatory indices in the overall cohort and according to psoriatic arthritis and special-site involvement during risankizumab treatment.

Index	Population/Subgroup	Baseline	Week 12	Week 24	Week 52	Week 104	Week 156	Week 208	Main Statistical Findings
**NLR**	Overall cohort	2.3 ± 1.5	2.0 ± 1.2	2.3 ± 1.7	2.2 ± 2.0	2.3 ± 2.5	2.0 ± 0.9	2.5 ± 2.1	No significant change from baseline
	Psoriatic arthritis	2.4 ± 1.3	2.1 ± 1.0	2.4 ± 2.8	2.3 ± 1.3	2.0 ± 0.9	2.0 ± 0.9	2.4 ± 1.1	No significant difference vs. patients without PsA
	Nail psoriasis	2.5 ± 1.6	2.1 ± 1.5	2.1 ± 1.0	2.7 ± 3.4	2.0 ± 0.5	2.4 ± 1.2	2.2 ± 1.1	No significant difference vs. patients without nail psoriasis
	Genital psoriasis	2.2 ± 1.5	2.3 ± 1.7	2.0 ± 0.9	2.5 ± 3.6	2.2 ± 1.1	2.0 ± 0.9	2.3 ± 1.5	**Week 52: change +0.4 vs. −0.2; nominal *p* = 0.036**
**PLR**	Overall cohort	130.3 ± 64.5	123.1 ± 62.8	117.9 ± 53.9	118.2 ± 81.4	119.1 ± 69.3	116.2 ± 56.5	112.6 ± 58.6	**Week 24: nominal *p* = 0.021; pBonf = 0.125. Week 52: nominal *p* = 0.004; pBonf = 0.024**
	Psoriatic arthritis	132.2 ± 51.7	131.1 ± 62.7	119.5 ± 56.7	126.6 ± 51.4	121.1 ± 41.0	113.7 ± 40.3	133.9 ± 47.1	**Week 208: change +5.1 vs. −36.0; nominal *p* = 0.036**
	Nail psoriasis	131.2 ± 77.4	115.5 ± 39.6	119.1 ± 51.8	135.7 ± 131.5	112.1 ± 48.1	128.8 ± 79.5	83.1 ± 81.3	No significant difference vs. patients without nail psoriasis
	Genital psoriasis	128.8 ± 90.4	130.9 ± 60.7	121.3 ± 53.2	129.0 ± 138.9	116.4 ± 62.0	131.8 ± 72.5	104.8 ± 69.4	**Week 52: change +3.9 vs. −18.4; nominal *p* = 0.033**
**MLR**	Overall cohort	0.3 ± 0.2	0.3 ± 0.3	0.3 ± 0.2	0.3 ± 0.1	0.3 ± 0.2	0.3 ± 0.2	0.4 ± 0.2	No significant change from baseline
	Psoriatic arthritis	0.3 ± 0.2	0.3 ± 0.1	0.3 ± 0.2	0.3 ± 0.1	0.3 ± 0.2	0.3 ± 0.1	0.3 ± 0.2	No significant difference vs. patients without PsA
	Nail psoriasis	0.3 ± 0.2	0.4 ± 0.5	0.3 ± 0.1	0.3 ± 0.2	0.3 ± 0.1	0.4 ± 0.3	0.5 ± 0.2	No significant difference vs. patients without nail psoriasis
	Genital psoriasis	0.3 ± 0.2	0.3 ± 0.1	0.3 ± 0.1	0.3 ± 0.2	0.3 ± 0.1	0.3 ± 0.2	0.4 ± 0.2	No significant difference vs. patients without genital psoriasis
**SII**	Overall cohort	602.1 ± 596.9	501.7 ± 325.7	543.1 ± 374.7	602.2 ± 1200.5	553.4 ± 557.1	486.7 ± 263.9	559.0 ± 488.2	No significant change from baseline
	Psoriatic arthritis	631.7 ± 465.9	518.4 ± 311.8	570.8 ± 567.0	586.3 ± 299.6	497.2 ± 209.2	461.0 ± 188.0	565.5 ± 276.6	No significant difference vs. patients without PsA
	Nail psoriasis	693.2 ± 884.0	477.8 ± 266.0	505.0 ± 257.7	912.3 ± 2253.5	480.8 ± 190.2	581.8 ± 366.2	396.7 ± 338.3	**Week 24: change −249.4 vs. +21.2; nominal *p* = 0.049**
	Genital psoriasis	625.1 ± 968.0	542.6 ± 359.4	471.7 ± 244.7	854.4 ± 2388.1	524.7 ± 435.9	515.2 ± 364.4	514.7 ± 420.7	**Week 52: change +234.1 vs. −68.1; nominal *p* = 0.025**
**SIRI**	Overall cohort	1.4 ± 1.2	1.3 ± 1.5	2.0 ± 3.2	3.1 ± 14.8	2.0 ± 3.7	1.8 ± 3.0	1.9 ± 1.6	**Week 24: nominal *p* = 0.022; pBonf = 0.133**
	Psoriatic arthritis	1.6 ± 1.3	1.0 ± 0.5	2.1 ± 3.5	1.8 ± 2.0	1.2 ± 0.8	1.5 ± 1.4	1.5 ± 0.8	No significant difference vs. patients without PsA
	Nail psoriasis	1.6 ± 1.2	1.5 ± 1.5	2.1 ± 3.8	7.1 ± 28.0	2.3 ± 3.5	2.1 ± 2.6	2.5 ± 1.5	**Week 208: change +1.5 vs. −0.1; nominal *p* = 0.038 (n = 6)**
	Genital psoriasis	1.4 ± 0.9	1.2 ± 0.9	1.6 ± 3.3	2.4 ± 5.5	2.0 ± 3.4	1.2 ± 0.8	2.1 ± 1.5	No significant difference vs. patients without genital psoriasis

Data are presented as mean ± standard deviation. The number of patients with available laboratory data varies across time points. Overall cohort: n = 250; psoriatic arthritis: 59/248 (23.8%); nail psoriasis: 72/246 (29.3%); genital psoriasis: 54/247 (21.9%). For the overall cohort, nominal *p*-values derive from paired *t*-tests of change from baseline; Bonferroni-adjusted *p*-values for the six repeated visits are reported for findings highlighted in the table, consistent with the primary longitudinal analysis. For each clinical subgroup, nominal *p*-values compare changes from baseline between patients with and without the corresponding manifestation. These are not direct comparisons among four independent groups, and the subgroups may overlap. **Abbreviations**: NLR, neutrophil-to-lymphocyte ratio; PLR, platelet-to-lymphocyte ratio; MLR, monocyte-to-lymphocyte ratio; SII, systemic immune-inflammation index; SIRI, systemic inflammation response index; PsA, psoriatic arthritis.

**Table 5:** Number of patients with nail and genital psoriasis at baseline and at the various follow-up time points.

Population/Subgroup	Baseline	Week 12	Week 24	Week 52	Week 104	Week 156	Week 208
Overall cohort	234	107	132	143	105	76	43
Psoriatic arthritis	57	19	35	39	28	24	17
Nail psoriasis	71	33	39	39	22	15	6
Genital psoriasis	50	27	35	35	18	12	7

#### 3.6.2. Nail Psoriasis

Nail psoriasis was present in 72/246 patients with available information (29.3%). In this subgroup, NLR and PLR showed non-linear fluctuations, whereas MLR remained generally stable. At week 24, SII decreased more markedly in patients with nail involvement than in those without it, with mean changes of −249.4 and +21.2, respectively (*p* = 0.049). This difference was not confirmed at subsequent visits. SIRI showed higher mean values at weeks 52 and 208; however, a nominal significant between-group difference was observed only at week 208 (n = 6), with mean changes of +1.5 in patients with nail psoriasis and −0.1 in those without nail involvement (*p* = 0.038).

#### 3.6.3. Genital Psoriasis

Genital psoriasis was present in 54/247 patients with available information (21.9%). Significant differences were confined to week 52. At this visit, changes in NLR, PLR, and SII differed significantly from those observed in patients without genital involvement: +0.4 versus −0.2 for NLR (*p* = 0.036), +3.9 versus −18.4 for PLR (*p* = 0.033), and +234.1 versus −68.1 for SII (*p* = 0.025). These differences were not maintained at weeks 104, 156, or 208. No significant between-group differences were observed for MLR or SIRI at any time point.

## 4. Discussion

### 4.1. Principal Findings

The DERMIS study shows that, over up to four years of risankizumab treatment, routine complete blood count-derived inflammatory indices do not provide reliable, objective, and reproducible surrogates of cutaneous response. Risankizumab produced a marked and sustained improvement in PASI, whereas NLR, MLR, SII, and SIRI showed stable or fluctuating trajectories without a consistent correlation with PASI. PLR displayed the most consistent downward pattern, with nominal reductions at weeks 24 and 52; after Bonferroni correction, the reduction at week 52 remained statistically significant, whereas the week-24 reduction did not. This isolated adjusted finding supports PLR as the most promising of the evaluated indices, but the absence of a consistent association across all visits and the lack of correlation with PASI argue against considering it a validated monitoring biomarker. These findings suggest that the systemic inflammatory information captured by routine hematological ratios is not interchangeable with the clinical inflammatory activity captured by PASI.

The divergence between the durable PASI response and the heterogeneous biomarker trajectories is clinically important. It indicates that improvement in visible psoriatic plaques after IL-23 blockade does not necessarily imply proportional normalization of peripheral blood inflammatory indices. These indices are biologically plausible but non-specific; they are influenced by age, infections, smoking, medications, obesity, cardiometabolic comorbidities, trauma, and other inflammatory or hematological perturbations [28,29,30,31,32,33]. In a real-world cohort, such confounders may be sufficient to dilute or obscure a treatment-related signal.

### 4.2. Comparison with Studies of Psoriasis Severity and Baseline Inflammation

Several studies have supported a relationship between blood count-derived indices and psoriasis presence or severity. Yorulmaz et al. reported higher SII levels in patients with psoriasis compared with controls as well as higher SII values in moderate-to-severe disease and psoriatic arthritis [10]. Dincer Rota and Tanacan similarly found higher SII values in psoriasis and proposed a cut-off value for disease activation [11]. Solak and Kara reported that SII, SIRI, NLR, monocyte-to-HDL ratio, and C-reactive protein were higher in psoriasis patients, with several indices correlating with PASI [15]. Similar associations were reported by Zhang et al., who found higher NLR, MLR, PLR, MHR, and SII in psoriasis and observed positive correlations between PASI and NLR, MLR, MHR, and SII [14].

Population-based studies have extended this signal beyond clinical cohorts. Zhao et al. and Ding et al. used National Health and Nutrition Examination Survey data to show associations between elevated SII and psoriasis risk [16,17]. Furthermore, a machine-learning analysis suggested that inflammatory indices contribute to psoriasis classification, although performance was moderate and the most informative markers differed across models [34]. Overall, these studies support the concept that blood count-derived indices capture part of the inflammatory milieu associated with psoriasis. However, most of them are cross-sectional, and their findings do not automatically imply that the same indices can track treatment response in individual patients over time.

### 4.3. Comparison with Biological Treatment Studies

Published treatment studies have yielded heterogeneous results. Kulakli et al. reported significant decreases in NLR, PLR, MLR, SII, SIRI, and C-reactive protein after 12 weeks of biological therapy, with low but significant correlations between PASI reduction and PLR, SII, and SIRI [19]. Tamer et al. showed significant decreases in SII and SIRI after three months of treatment with several biologics, including risankizumab, but also emphasized the lack of consensus on optimized biomarker use [20]. Morariu et al. found that multiple indices varied significantly during biological or small-molecule treatment and that baseline d-NLR and SIRI independently predicted super-responder status [21]. Similarly, Kimak-Pielas et al. reported significant decreases in NLR, PLR, SII, and C-reactive protein over 18 months of biological therapy, with no significant differences observed across anti-TNF, anti-IL-12/23, anti-IL-17, and anti-IL-23 classes [22].

Compared with previous studies of blood count-derived inflammatory markers during biological therapy [18,19,20,21,22,23,24], the DERMIS study differs in three key aspects. First, it focused exclusively on risankizumab rather than combining different biological classes or systemic drugs. Second, it included a larger risankizumab-treated cohort than previous risankizumab-specific reports. Third, the follow-up was extended to 208 weeks, allowing for the assessment of whether early changes persist over several years. Our observation that several indices decreased at week 12 but later fluctuated suggests that early immune modulation may be detectable but may not remain stable once cutaneous inflammation is controlled and other determinants of systemic inflammation become relatively more influential.

The closest comparison is the prospective multicenter 52-week risankizumab study by Di Cesare et al., which reported progressive reductions in CRP, ESR, NLR, SII, and SIRI, especially in very early responders [18]. However, the DERMIS study did not reproduce a strong association between early PASI90 response and inflammatory-index modulation. This difference might be explained by study design and population structure. Di Cesare et al. analyzed 103 risankizumab responders with a median age of 54 years and excluded patients with incomplete data or those who discontinued before week 52. In contrast, the DERMIS study included a broader, older, and more heterogeneous real-world population, utilizing available-case analyses and including patients who had incomplete long-term data. In such a setting, comorbidities and non-psoriatic inflammatory stimuli may obscure a responder-specific biomarker profile.

### 4.4. IL-23 Inhibition, PLR, and the Role of Platelets

PLR deserves specific consideration. In DERMIS, PLR was the only index showing a relatively consistent downward trend with nominal reductions at weeks 24 and 52. After Bonferroni correction, the week-52 reduction remained statistically significant, whereas the week-24 reduction did not. The week-52 completer and week-104 completer analyses also retained a significant PLR reduction at week 52 after correction, and the week-52 completer cohort additionally retained significance at week 24. These findings strengthen the consistency of the PLR signal while remaining exploratory. The observation is biologically plausible because platelets are active regulators of inflammatory and vascular processes, including leukocyte recruitment, endothelial activation, and atherosclerosis [35]. Platelets have also been implicated in psoriatic inflammation and cardiometabolic risk [35]. A post hoc analysis of VOYAGE I/II and ECLIPSE found that guselkumab reduced NLR, PLR, and MLR compared with placebo and that reductions were not simply explained by skin response [25]. A long-term guselkumab study reported significant reductions in PLR and SIRI at week 156, while correlations with PASI were weak [26].

Taken together with these data, the DERMIS PLR signal may indicate that platelet-related inflammatory pathways are less susceptible to confounding proinflammatory factors and, therefore, more sensitive than neutrophil- or monocyte-weighted ratios to long-term IL-23 inhibition. While this interpretation remains speculative, and PLR should not yet be considered a validated monitoring biomarker, among the indices analyzed in the DERMIS study it appears to be the most suitable candidate for prospective validation.

### 4.5. SII, SIRI, NLR, and MLR: Why the Signal Was Not Stable

NLR, SII, and SIRI all include neutrophil count in their numerator, which may partly explain their instability. Neutrophils are highly reactive to acute and chronic stimuli not directly related to psoriasis, including smoking, medications such as lithium, testosterone-related effects, atherosclerosis, and trauma [28,29,30,31,32]. SII and SIRI are more complex than NLR because they incorporate platelets or monocytes, but this complexity does not necessarily translate into greater specificity for psoriatic inflammation. In DERMIS, SII mirrored the fluctuating pattern of NLR, whereas SIRI showed an early decrease followed by a nominal increase at week 24 and later fluctuations; the week-24 SIRI signal did not remain significant after Bonferroni correction. These behaviours argue against their use as stand-alone markers of risankizumab response in routine practice.

MLR was the most stable parameter. This observation is consistent with several studies suggesting that IL-23 inhibitors do not consistently influence MLR [18,25,26]. Stability may appear attractive for a biomarker, but in this setting, it suggests limited sensitivity to therapeutic modulation. Therefore, MLR should not be prioritized as an endpoint in future risankizumab monitoring studies unless embedded in broader immune signatures.

### 4.6. BMI and Metabolic Phenotype

BMI was included as a secondary endpoint because obesity is a major determinant of low-grade systemic inflammation and may interact with the IL-23/Th17 axis. Adipose tissue is an active endocrine and immune organ capable of producing adipokines and inflammatory mediators, including leptin, which may promote Th17-related inflammation [5,36]. In the DERMIS study, patients with obesity tended to have higher PASI values and higher inflammatory indices at several time points, although between-group differences were inconsistent. A potentially relevant exploratory finding was the tendency towards a reduction in some inflammatory indices in patients with obesity during long-term follow-up, whereas values in normal-weight and overweight groups remained largely stable or increased.

One possible explanation is that patients with obesity start from a higher inflammatory baseline and may, therefore, have greater scope for systemic improvement during IL-23 blockade. Another possibility is that IL-23 inhibition exerts distinct systemic effects according to metabolic phenotype. Bardazzi et al. reported that obesity was associated with the most pronounced decrease in SII and SIRI during biological therapy in a comorbidity-enriched psoriasis cohort [23]. However, the DERMIS study did not confirm a consistent statistically significant BMI effect, and the significant findings at week 208 were limited by small subgroup sizes. Dedicated prospective studies in patients with obesity, with detailed metabolic characterization, are needed.

### 4.7. Psoriatic Arthritis and Special-Site Involvement

Overall, the presence of psoriatic arthritis, nail psoriasis, or genital psoriasis did not identify a stable or reproducible longitudinal trend of blood count-derived inflammatory indices during risankizumab treatment. Statistically significant findings were isolated, inconsistent across markers and time points, and in some cases based on small subgroups. These clinical manifestations were not mutually exclusive, therefore patients within the overall cohort could belong to multiple subgroups. Accordingly, the statistical analyses compared patients with and without each manifestation separately and should not be interpreted as a direct comparison among four independent groups. Given the multiple testing burden, these secondary subgroup findings are hypothesis-generating only.

### 4.8. Strengths and Limitations

The main strengths of the DERMIS study include its large single-drug cohort, multicenter real-world design, and long observation period of up to 208 weeks. This design allowed evaluation of whether early changes in inflammatory indices persist over the long term and provided a clinically realistic picture of biomarker dynamics in patients treated with a highly effective anti-IL-23 monoclonal antibody. The study also explored the influence of BMI, sex, cardiometabolic comorbidities, and previous biological exposure, variables that are underrepresented in previous biomarker studies.

Several limitations should be acknowledged. First, the retrospective, non-randomized design precluded comprehensive control of potential confounders. Smoking, physical activity, alcohol consumption, intercurrent infections, trauma, concomitant medications, and changes in body weight were not systematically recorded and therefore could not be formally adjusted for. Although this reflects routine real-world clinical practice, it limits causal interpretation of the observed biomarker trajectories.

Second, data availability decreased substantially at longer follow-up, with only 43 of 250 patients (17.2%) contributing data at week 208. This reduced statistical precision and power, particularly for subgroup analyses, and may have introduced selection or attrition bias. Although the available-case approach allowed inclusion of all available real-world observations, it also resulted in varying sample sizes across time points.

Finally, the large number of longitudinal and subgroup comparisons increased the risk of type I error, particularly for analyses involving small subgroups at later visits. To address this issue, Bonferroni correction was applied separately to the six repeated comparisons within each parameter and analysis table. Nevertheless, these analyses were exploratory and the study was not specifically powered for multiple subgroup comparisons. Accordingly, isolated findings, particularly those observed at week 208, should be interpreted cautiously and require confirmation in adequately powered prospective studies.

## 5. Conclusions

In this multicenter real-world study, risankizumab demonstrated high and sustained clinical efficacy in patients with cutaneous psoriasis, with significant reductions in PASI scores throughout the follow-up period. However, the longitudinal trajectories of NLR, MLR, SII, and SIRI values were heterogeneous and did not provide a reliable surrogate marker of cutaneous response. MLR remained substantially stable and appears to have very limited utility as a monitoring biomarker in risankizumab-treated patients. Although NLR, SII, and SIRI may retain biological relevance, their evaluation requires prospective studies specifically designed to address confounding factors and prognostic value. PLR was the only index to show a relatively consistent downward trend and it appears to be the most promising candidate for future validation. BMI may modulate systemic inflammatory indices over the long term, particularly in patients with obesity, while sex, cardiometabolic comorbidities, and previous biological exposure did not show a consistent influence. Overall, the DERMIS study supports a cautious interpretation of blood count-derived inflammatory indices: although cost-effective and biologically meaningful, they should not currently replace clinical assessment or be used as stand-alone biomarkers of response to risankizumab.

## Figures and Tables

**Table 1:** Baseline demographic and clinical characteristics of the DERMIS cohort.

Characteristic	Overall Cohort
**Study population**	
Participants, N	250
Recruiting centers, n	10
**Age, years**	
Mean ± SD	56.6 ± 14.5
Median (Q1–Q3)	56.5 (47.0–68.0)
Range	22–89
**Age at psoriasis diagnosis, years**	
Available data, n/N	248/250
Mean ± SD	18.2 ± 12.3
Median (Q1–Q3)	16.5 (9.0–25.0)
Range	1–71
**Sex**	
Female, n/N (%)	150/249 (60.2)
Male, n/N (%)	99/249 (39.8)
Missing, n/N (%)	1/250 (0.4)
**Body mass index**	
BMI, kg/m^2^, mean ± SD	27.3 ± 5.0
BMI, kg/m^2^, median (Q1–Q3)	26.5 (23.8–30.1)
BMI, kg/m^2^, range	17.5–45.8
Normal weight or underweight (<25 kg/m^2^), n (%)	94 (37.6)
Overweight (25 to <30 kg/m^2^), n (%)	93 (37.2)
Obesity (≥30 kg/m^2^), n (%)	63 (25.2)
**Previous exposure to biological therapy**	
Biologic-experienced, n/N (%)	173/249 (69.5)
Biologic-naïve, n/N (%)	76/249 (30.5)
Missing, n/N (%)	1/250 (0.4)
**Psoriasis characteristics**	
Psoriatic arthritis, n/N (%)	59/248 (23.8)
Nail psoriasis, n/N (%)	72/246 (29.3)
Genital psoriasis, n/N (%)	54/247 (21.9)
**Comorbidities**	
Hypertension, n/N (%)	99/248 (39.9)
Hepatic steatosis, n/N (%)	54/249 (21.7)
Diabetes mellitus, n/N (%)	44/249 (17.7)
Coronary artery disease, n/N (%)	38/249 (15.3)
Depression, n/N (%)	29/248 (11.7)
**Baseline PASI**	
Available data, n/N (%)	234/250 (93.6)
Mean ± SD	12.8 ± 7.3
Median (Q1–Q3)	11.4 (9.0–16.0)
Range	0–53

Data are presented as n (%), mean ± standard deviation, or median (first–third quartile), as appropriate. Percentages for variables with missing data were calculated using the number of patients with available information as the denominator. Previous biological exposure was derived from the database variable “condition” (1 = biologic-experienced; 0 = biologic-naive). BMI, body mass index; PASI, Psoriasis Area and Severity Index; Q1, first quartile; Q3, third quartile; SD, standard deviation.

**Table 2:** Longitudinal changes in systemic inflammatory indices during risankizumab treatment.

	Baseline (n = 234)	Week 12 (n = 107)	Week 24 (n = 132)	Week 52 (n = 143)	Week 104 (n = 105)	Week 156 (n = 76)	Week 208 (n = 43)
**NLR** **Neutrophil-to-lymphocyte ratio** (*p*-value nominal) * [*p*-value Bonferroni] **	2.3 ± 1.5	2.0 ± 1.2 (0.514) [1.000]	2.3 ± 1.7 (0.940) [1.000]	2.2 ± 2.0 (0.640) [1.000]	2.3 ± 2.5 (0.367) [1.000]	2.0 ± 0.9 (0.359) [1.000]	2.5 ± 2.1 (0.623) [1.000]
**PLR** **Platelet-to-lymphocyte ratio** (*p*-value nominal) * [*p*-value Bonferroni] **	130.3 ± 64.5	123.1 ± 62.8 (0.615) [1.000]	117.9 ± 53.9 **(0.021)** [0.125]	118.2 ± 81.4 **(0.004)** **[0.024]**	119.1 ± 69.3 (0.587) [1.000]	116.2 ± 56.5 (0.200) [1.000]	112.6 ± 58.6 (0.067) [0.399]
**MLR** **Monocyte-to-lymphocyte ratio** (*p*-value nominal) * [*p*-value Bonferroni] **	0.3 ± 0.2	0.3 ± 0.3 (0.909) [1.000]	0.3 ± 0.2 (0.401) [1.000]	0.3 ± 0.1 (0.369) [0.335]	0.3 ± 0.2 (0.555) [1.000]	0.3 ± 0.2 (0.300) [1.000]	0.4 ± 0.2 (0.893) [1.000]
**SII** **Systemic immune-inflammation index** (*p*-value nominal) * [*p*-value Bonferroni] **	602.1 ± 596.9	501.7 ± 325.7 (0.184) [1.000]	543.1 ± 374.7 (0.309) [1.000]	602.2 ± 1200.5 (0.923) [1.000]	553.4 ± 557.1 (0.509) [1.000]	486.7 ± 263.9 (0.350) [1.000]	559.0 ± 488.2 (0.371) [1.000]
**SIRI** **Systemic inflammation response index** (*p*-value nominal) * [*p*-value Bonferroni] **	1.4 ± 1.2	1.3 ± 1.5 (0.980) [1.000]	2.0 ± 3.2 **(0.022)** [0.133]	3.1 ± 14.8 (0.157) [0.944]	2.0 ± 3.7 (0.061) [0.369]	1.8 ± 3.0 (0.142) [0.849]	1.9 ± 1.6 (0.530) [1.000]

Data are presented as mean ± SD. Statistically significant *p*-values are shown in bold. * Paired *t*-test *p*-values refer to within-subject comparisons against baseline. ** For each parameter, the Bonferroni correction was applied to the six comparisons against baseline.

## Data Availability

Data are available upon reasonable request.
